# Using niche-modelling and species-specific cost analyses to determine a multispecies corridor in a fragmented landscape

**DOI:** 10.1371/journal.pone.0183648

**Published:** 2017-08-25

**Authors:** Karen E. DeMatteo, Miguel A. Rinas, Juan Pablo Zurano, Nicole Selleski, Rosio G. Schneider, Carina F. Argüelles

**Affiliations:** 1 Washington University in St. Louis, Department of Biology & Environmental Studies, St. Louis, Missouri, United States of America; 2 WildCare Institute at the Saint Louis Zoo, St. Louis, Missouri, United States of America; 3 University of Missouri, Division of Biological Sciences, Columbia, Missouri, United States of America; 4 Ministerio de Ecología y Recursos Naturales Renovables, Posadas, Misiones, Argentina; 5 Facultad de Ciencias Exactas, Químicas y Naturales, Departamento de Genética, Universidad Nacional de Misiones (UNaM), Posadas, Misiones, Argentina; 6 Grupo de Investigación en Genética Aplicada (GIGA), IBS – Nodo Posadas, UNaM – CONICET, Posadas, Misiones, Argentina; Charles University, CZECH REPUBLIC

## Abstract

Misiones, Argentina, contains the largest remaining tract of Upper Paraná Atlantic Forest ecoregion; however, ~50% of native forest is unprotected and located in a mosaic of plantations, agriculture, and pastures. Existing protected areas are becoming increasingly isolated due to ongoing habitat modification. These factors, combined with lower than expected regional carnivore densities, emphasize the need to understand the effect of fragmentation on animal movement and connectivity between protected areas. Using detection dogs and genetic analyses of scat, we collected data on jaguars (*Panthera onca*), pumas (*Puma concolor*), ocelots (*Leopardus pardalis*), oncillas (*Leopardus tigrinus*), and bush dogs (*Speothos venaticus*) across habitats that varied in vegetation, disturbance, human proximity, and protective status. With MaxEnt we evaluated habitat use, habitat suitability, and potential species richness for the five carnivores across northern-central Misiones, Argentina. Through a multifaceted cost analysis that included unique requirements of each carnivore and varying degrees of overlap among them, we determined the optimal location for primary/secondary corridors that would link the northern-central zones of the Green Corridor in Misiones and identified areas within these corridors needing priority management. A secondary analysis, comparing these multispecies corridors with the jaguar’s unique requirements, demonstrated that this multispecies approach balanced the preferences of all five species and effectively captured areas required by this highly restricted and endangered carnivore. We emphasize the potential importance of expanding beyond a single umbrella or focal species when developing biological corridors that aim to capture the varied ecological requirements of coexisting species and ecological processes across the landscape. Detection dogs and genetic analyses of scat allow data on multiple species to be collected efficiently across multiple habitat types independent of the degree of legal protection. These data used with multifocal GIS analyses balance the varying degree of overlap and unique properties among them allowing for comprehensive conservation strategies to be developed relatively rapidly. Our comprehensive approach serves as a model to other regions faced with habitat loss and lack of data. The five carnivores focused on in our study have wide ranges, so the results from this study can be expanded and combined with surrounding countries, with analyses at the species or community level.

## Introduction

The loss and conversion of native habitat have resulted in many protected areas becoming increasingly isolated, forcing species to navigate a matrix of habitats. These movements threaten the long-term survival of species requiring large ranges to meet ecological and energetic needs as they are inevitably exposed to human-wildlife conflicts. Geographic Information Systems (GIS) have been used to determine corridors that minimize the cost of these movements [1,2]; however, the type of data used to construct these models and how decisions are made to select areas are still evolving in conservation biology.

One approach to maintaining connectivity across a landscape is to expand the existing network of protected areas [3,4,5,6]; however, this action can involve some complications including the removal of private land owners and the need for additional personnel to monitor these areas. In other cases, existing protected areas could be used as a series of “stepping stones” across the heterogeneous landscape where species spend less time in the intermediary areas versus actual protected areas. Though the fact that some species apparently avoid human disturbed areas (e.g., jaguar) [7,8] may mean these intermediary areas are physical barriers and prevent movement among the “stones”. Another approach is to identify intermediary areas with habitat that species find suitable and create corridors based on them. This could minimize the total amount of “new” area needed in the corridor by having existing protected areas act as “stepping stones” across the landscape [9].

Many corridors have been designed to optimize the movement of a single, typically large-bodied, focal species with the goal of having an umbrella effect on protecting coexisting species; however, research suggests that this targeted approach may fail to completely capture the varied ecological requirements of coexisting species and ecological processes across the landscape [10,11]. To overcome these limitations, an alternative modelling approach includes multiple species that share similar ecological requirements and sensitivity to human disturbance [12,13], although this may generate results that underestimate connectivity for species highly restricted in their movements. Therefore, a more comprehensive approach that balances the trade-offs that face single and multispecies corridors is to develop models that account for variation across multiple species and use information on the most restricted species to weight final decisions [10,11].

Another key point in these studies is to decide how to predict connectivity; specifically, the optimal locations and dimensions for corridors or wildlife linkages. While potential approaches are broad, including circuit theory [14], network flow [15], least-cost path (LCP), and least-cost corridor (LCC) [16,17,18], all aim to determine the least ecological cost for an organism to move between defined locations. Which approach is the best may depend on the study’s scale or focal species, but even on those specific levels there is a continuing debate about the methods. Both LCP and LCC look to balance varying resistances across a travel surface and identify the lowest cumulative resistance between assigned source and destination locations. While a LCP is always one pixel wide, the width of a LCC depends on how many paths between the source and destination locations share the same least accumulated cost. The optimal or minimal width for setting a corridor is also still being debated; however, it is clear that length and width must be balanced [10,19] and it has been suggested that width likely gains importance as corridor length increases [20].

In addition, one must decide which factors should be included in generating this resistance surface and the specific weights that should be assigned to reflect their effect (positive or negative) on animal movement [10,21]. A common method is to use resource selection function to weight the cost of movement across a matrix of habitats [22,23,24], which requires the analyst to convert resources selection or habitat preferences into a cost of movement across a surface [20]. When actual movement data is not available, one must default to using other strategies including species occurrence, density estimates, literature, and expert opinions [25,26,27]. Which measure is correct or more appropriate remains under discussion and may vary with species or conditions.

**Fig 1:**
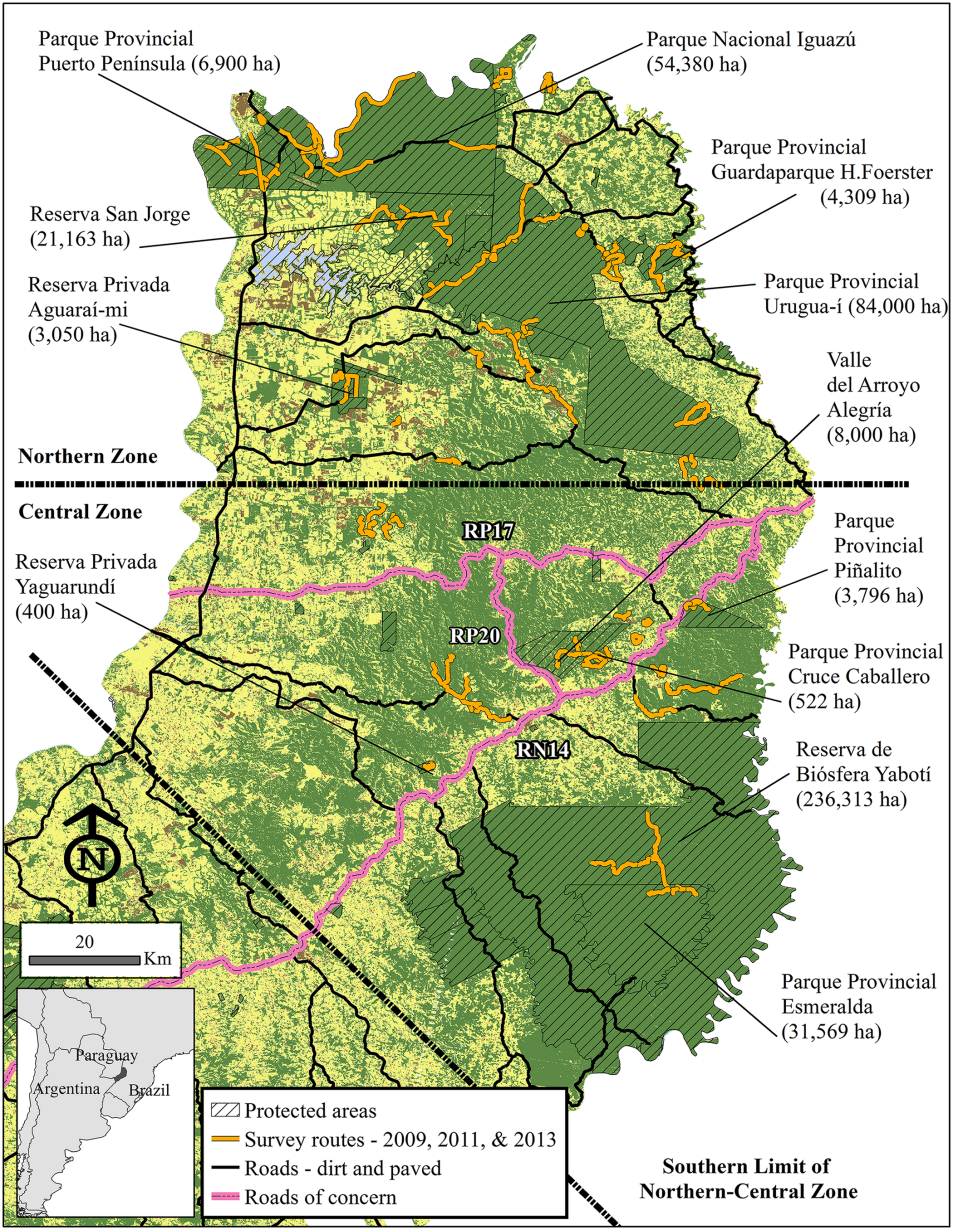
Map of northern-central Misiones with all survey routes shown relative to protected areas, roads, and the land-use pattern existing in Misiones in 2009. Land cover map reflects data from 2009 [35]. The largest protected areas and three roads of concern are labeled. RP17, RP20, and RN14 are highlighted because they are all located in areas where habitat conversion is ongoing, rural populations are expanding, and the roads themselves are being widen and converted from dirt to paved asphalt.

Small remnants of Upper Paraná Atlantic forest ecoregion (i.e., Green Corridor) still exist in Brazil and Paraguay, but in Argentina the province of Misiones contains the largest remaining tract of almost 1.4 million hectares. While 67% of this native forest is contained in northern-central (N-C) zones of Misiones, only 46.1% (429,998.3 ha) is protected in a series of areas that vary in size, adjacency, and degree of protection. This means that over 50% (503,218.8 ha) of the native forest is unprotected and located in a matrix of plantations, agriculture, and pastures [8,28,29] (Fig 1). Ongoing habitat conversion, an expanding network of roads, and population growth in rural areas of Misiones put connectivity between the largest blocks of protected areas in the N-C zones at risk [30,31,32]. These facts, combined with the region’s lower than expected carnivore densities because of poaching and forest degradation [33], emphasize the need to determine how fragmentation is affecting connectivity [34] between existing protected areas via negative influences on the movements of wide-ranging carnivores.

This work aims to determine a multispecies corridor model that maximizes connectivity between the largest blocks of protected areas in the N-C zones of Misiones (“stepping stones”) and identify areas requiring priority management. The goal is to balance species-specific preferences [36,37] for the five carnivores [jaguar (*Panthera onca*), puma (*Puma concolor*), ocelot (*Leopardus pardalis*), oncilla (*Leopardus tigrinus*), and bush dog (*Speothos venaticus*)], while ensuring the most restricted species (jaguar) is effectively captured to develop a multispecies corridor. The ultimate goal for this multispecies model is to contribute to the biological conservation of these five carnivores and the coexisting biodiversity. The strength of our approach comes from using noninvasive techniques to effectively sample multiple species over varying habitat integrity, using species-specific habitat suitability based on anthropogenic change to assign costs to the corridor matrices, and developing a corridor that covered the breadth of a multispecies ecosystem yet captured the ecological requirements of the most restricted species. While independently none of these techniques are new, their combined application has the potential to serve as a model that can extend beyond the borders of Misiones, Argentina.

## Materials and methods

### Field surveys

Data from N-C zones of Misiones were collected during three surveys (2009, 2011, and 2013) conducted primarily during the cool season (May-August). The Ministerio de Ecología y Recursos Naturales Renovables of Misiones (MEyRNR) issued all general permits related to our project in the province, collection of samples in the multiple provincial parks, and export permits of genetic samples. The Administración de Parques Nacionales of Argentina issued permits related to collection of samples in the national park.

Survey tracks consisted of two-lane paved roads, 1–2 lane dirt roads, and trails (established and existing machete cut) through forest. The team surveyed a total of 198 unique routes and walked a total of 1,142.9 km [mean (SD) = 5.8 (3.2) km; range = 0.4–21.1 km per route; Fig 1]. In addition to protected areas of native forest, surveys covered unique habitats including private native forest, small-scale agriculture, monoculture plantations of pine and eucalyptus, small communities with subsistence agriculture, pastures, and human-occupied areas. Of the total distance walked, 37.0% (423.3 km) was located outside of protected areas.

All surveys were completed using the same detection dog-handler team [8,29,38]. Detection dogs eliminate dependence on visitation rate to a specific location (e.g., camera trap) and instead switches the focus to locating evidence (e.g., olfactory) associated with the species’ natural behavior and movement patterns. Even in the rugged terrain of Misiones, Argentina, detection dogs have been able to effectively search large geographic areas and locate samples from multiple species, while ignoring samples from nontarget species that may be similar in their appearance or composition [8,29,38]. The training of the detection dog, swabbing of scat for genetic samples, collection of scat, and recording of field data were the same in all surveys [8,29,38].

### Genetic analyses

Scat swabs were processed using DNA extraction protocols and genetic analyses detailed in previous studies [8,29]; however, a brief summary is provided here. DNA was extracted from two independent swabs using a Qiagen (Venlo, Netherlands) DNeasy™ DNA extraction kit following a modified protocol by Vynne [39]. To identify species, a 110-bp (171-bp with primers) carnivore-specific region of mitochondrial cytochrome *b* gene (5′-AAACTGCAGCCCCTCAGAATGATATTTGTCCTCA-3′; 5′-TATTCTTTATCTGCCTATACATRCACG-3′ [40]) was amplified with a modified version of the protocols and reagents of Farrell [40] and Miotto [41]. Amplifications were performed on a MyCycler Thermal Cycler System (BioRad, Hercules, CA) in 25-μL volumes containing 2-μL DNA extract, 1× PCR Gold buffer [Applied Biosystems, Foster City, CA] 0.3-μM forward and reverse primer, 200 μM each dNTP, 5-mM MgCl_2_, 150-μg/mL BSA (Ambion^®^—Life Technologies, Grand Island, NY) and 1-U Ampli*Taq* Gold DNA polymerase (Applied Biosystems). The PCR profile consisted of 10-min denaturation at 95°C, followed by 40 cycles at 95°C for 30 s, 49°C for 45 s, 72°C for 45 s, and a final 30-min extension at 72°C. Purified PCR products were sequenced using the ABI PRISM BigDye Terminator v3.1 Cycle Sequencing Kits (ABI) and analyzed in an ABI 3100 Genetic Analyzer (ABI). Sequences were edited and aligned using Lasergene Seqman 8.1 (DNASTAR, Madison, WI) and compared with reference entries in GenBank using the Basic Local Alignment Search Tool (BLAST; [42]) to identify sequences from Neotropical species that had high similarity and closely-matched sample sequences.

Of the 917 total samples collected, species identity was confirmed in 761 (83.0%) (S1 Appendix). In the remaining scat swabs, species identity was not possible due to low quantity/quality DNA (n = 108, 11.8%) or urine contamination through scent marking animals (n = 48, 5.2%). While half of the samples were identified as oncilla (n = 494, 53.9%), there were 111 (12.1%) ocelot, 63 (6.9%) jaguar, 59 (6.4%) puma, and 34 (3.7%) bush dog. Exact sample locations are not reported or displayed per government request as a precaution to protect these threatened and endangered carnivores from targeted poaching.

### Modeling the ecological niche

MaxEnt 3.3.3.k was used to model ecological niches for the five carnivores and evaluate habitat suitability [43], as it has been reported to perform consistently better than other algorithms [44,45] and effective at working with small numbers of presence-only samples [45]. Models were restricted to the N-C zones in Misiones where data collection occurred, were fit using hinge features only that substantially improve model performance [46,47], and default regularization parameters [48]. Models were tested by randomly withholding 25% of presence localities for each species.

We generated a logistic output, which gives the probability of species presence on a scale of 0 to 1 and has been shown to improve model performance via model calibration of output values and corresponding suitability [48]. To convert the logistic output of each model to a binary prediction (habitat predicted suitable or unsuitable), a threshold or “cutoff” value was applied. All probability values equal or greater than the threshold were classified as suitable habitat with a high probability of species’ presence. While no standard rule applies to the use of extrinsic omission rate and proportional predicted area (proxy for commission rate) across datasets, we followed published recommendations for establishing criteria to evaluate both variables [43]. Specifically, our criteria were to have an omission rate of zero but set lower restrictions on the size of the potential predicted area. We compared the extrinsic omission rate and proportional predicted areas at several logistic thresholds [minimum training presence (MTP), fixed cumulative value (FCV), 10 percentile training presence (10PT), and maximum training sensitivity plus specificity (MTSS)] [49,50]. While MTP best fits this criterion in oncilla (0.007), FCV was determined to be the best fit in jaguar (0.008), puma (0.023), ocelot (0.028), and bush dog (0.014). While FCV was determined to be the less conservative choice in jaguar and puma, it was considered the better choice because it identified the maximum potential areas possible while still maintaining a zero-omission rate for both training and test data. Ecologically, these threshold values can be interpreted to contain cells that are predicted to be at least as suitable as those where the species was identified present.

### Predictor variables for the ecological niche model (ENM)

A neighborhood analysis, 30 m × 30 m resolution, was used to characterize conditions in neighborhood cells, such as habitat heterogeneity [51] (Table 1). Two neighborhood scales, 4 km and 7 km, were used to represent a range of home ranges from 50 km^2^ (potential minimum) to 150 km^2^. While the five species differ in home range sizes, this upper range has been reported for jaguars [52,53], pumas [54], and bush dogs [55]; however, the lower range (50 km^2^) is likely a better match for the maximum values of oncilla and ocelot [56]. The predictor variables chosen to help fine-tune the species-specific distributions fall into five categories: topographic, vegetative landscape, natural features, protection, and anthropogenic (Table 1) [7]. To balance the importance of protected areas in providing valuable habitat without skewing the model away from areas outside of protected areas, we included a single variable (7 km radius) related to legal protection afforded to habitat through their designation as a legal park or reserve (Table 1). Information directly related to roads (frequency or distance to) was not included, as we believed these would skew the model since a large portion of locations are closely associated with them. All predictor variables were converted into raster with 30 m × 30 m resolution. An initial model was run with all 22 predictor variables (Table 1). Evaluation of jackknife tests of variable importance and response curves for individual variables eliminated 10 variables from the final model due to either no effect or a negative effect on model performance (Table 1). This evaluation used the regularized training gain and test gain generated by MaxEnt, which accounts for dependency among predictor variables and compares the effect of a specific feature by itself with a model of all features except that single feature. Efficacy of the 12 selected predictor variables was evaluated by MaxEnt jackknife tests using test gain and area under the ROC curve (AUC) on test data, with the latter providing a threshold-independent measure of overall model accuracy [57].

**Table 1:** Summary of predictor variables tested and used in the species-specific ecological niche models. List includes all 22 predictor variables used in the development of the species-specific ecological niche models and whether the variable was used in the final model. A total of 12 predictor variables were used in the final models. For each variable, there is a description of what it represents, the original data source, and any calculations used to generate it.

Variable	Final model	Description	Data source	Methods of Calculation
**Topographic:**				
**Elevation**	no	Elevation above sea level obtained from ASTER Global Digital Elevation (GDEM) Model V003 (AST14DEM3).	GDEM is a product of NASA and METI. NASA LP DAAC. (2001). ASTER DEM Product [Data set]. NASA LP DAAC. Available from: https://doi.org/10.5067/aster/ast14dem.003ASTER.).	
**Slope**	no	Terrain slope expressed in degrees.		Calculated from Elevation grid using ArcMap 10.4.
**Vegetative landscape:**				
**Land use type**	yes	Habitat type represented in each cell.	Derived using 30 m × 30 m land cover raster grids of Misiones, Argentina from 2009 as described in [35].	
**Forest (r4)**	yes	Frequency of cells occupied by native forest in a circle of 4-km (r4) radius around the focal cell.	See [35].	Calculated from ‘Land use type’ using *Neighborhood–Focal Statistics* and *Raster Calculator* of Spatial Analyst for ArcMap 10.4 (Focal Statistics–ArcMap).
**Forest (r7)**	no	Frequency of cells occupied by native forest in a circle of 7-km (r7) radius around the focal cell.	See [35].	Focal Statistics–ArcMap
**Agriculture (r4 and r7)**	yes	Frequency of cells occupied by agriculture in a circle of 4-km (r4) and 7-km (r7) radius around the focal cell.	See [35].	Focal Statistics–ArcMap
**Pastures (r4 and r7)**	yes	Frequency of cells occupied by pastures in a circle of 4-km (r4) and 7-km (r7) radius around the focal cell.	See [35].	Focal Statistics–ArcMap
**Plantations (r4 and r7)**	yes	Frequency of cells occupied by monoculture tree plantations in a circle of 4-km (r4) and 7-km (r7) radius around the focal cell.	See [35].	Focal Statistics–ArcMap
**Landscape heterogeneity (r4 and f7)**	yes	Landscape heterogeneity index of the focal cell calculated in a circle of 4-km (r4) and 7-km (r7) radius around the focal cell. The index is based on the Shannon-Wiener diversity index of habitat type diversity, where higher heterogeneity values represent strongly anthropogenic landscapes (or a greater number of human modified landscapes in a small area).	See [35].	Focal Statistics–ArcMap
**Natural features:**				
**Rivers (r4 and r7)**	no	Frequency of cells occupied by rivers in a circle of 4-km (r4) and 7-km (r7) radius around the focal cell.	See [35].	Focal Statistics–ArcMap
**Rivers distance**	no	Straight line distance to the closest river.	Geocommunity GIS Data Depot. Available from: http://data.geocomm.com.	Calculated using *Distance—Euclidean Distance* of Spatial Analyst for ArcMap 10.4 (Euclidean Distance–ArcMap).
**Protection:**				
**Protection (r7)**	no	Mean value of relative protection in the cells inside a circle of 7-km (r7) radius around the focal cell. Five categories of protection were defined: the oldest and best protected national park (value = 100), other national parks and national reserves (value = 80), provincial/state parks (value = 60), private reserves (value = 40), and multiple use reserves (value = 20). Reserves not officially implemented were not included.		Focal Statistics–ArcMap
**Anthropogenic:**				
**Cost of access**	no	Accessibility cost for humans measured as the hours needed to access the focal cell from the nearest town or city.	Layer based on accessibility cost used published in [7] but modified to include protected areas as a movement barrier and resampled in ArcMap 10.4 for 30 m× 30 m.	
**Urban (r4 and r7)**	yes	Frequency of cells occupied by urbanized areas in a circle of 4-km (r4) and 7-km (r7) radius around the focal cell.	See [35].	Focal Statistics–ArcMap
**Urban distance**	no	Straight line distance to the closest town or city.	Instituto Nacional de Estadística y Censos (INDEC). Available from: http://www.indec.gob.ar/nivel2_default.asp?id_tema=1&seccion=T.	Euclidean Distance–ArcMap
**Rural population density**	no	Rural population density per department as obtained from the national census (2001).	Instituto Nacional de Estadística y Censos (INDEC). Available from: http://www.indec.gob.ar/nivel2_default.asp?id_tema=2&seccion=P	

### Potential species richness (PSR)

The PSR identifies areas where none (value = 0) or all (value = 5) of the carnivores overlap in habitat defined as suitable [58]. PSR, which is obtained by combining the five species-specific ENMs, highlights areas where multiple species have suitable habitat and quantifies species number. This degree of overlap is an additional method to confirm range-restricted species and identify areas where habitat restoration could result in increased PSR.

### Cost analyses

Each species-specific resistance surface for the cost analyses was developed in ArcGIS 10.4 (ESRI) using the 12 raster grids that generated the individual ENMs (Table 1). For each grid, the extent was fit using the previously defined threshold of the species-specific ENM, with the outside of this extent classified as unsuitable habitat, a lower probability of species’ presence, and restricted movement in the cost analysis. This conservative approach is linked back to the previously discussed debate on how to apply thresholds to potential distribution modeling and cost analyses (e.g., [10,20,21,22,25,49]). After trying several approaches with the data, we identified an appropriate model for predicting potential connectivity outside of protected areas for our study by combining field data, knowledge of ongoing land conversion outside of protected areas, and setting areas outside of the predicted species distribution to regions with restricted movement.

To maximize variability across the cost matrix, each grid was reclassified into 10 divisions. While equal interval breaks were applied to the categorically divided land use data, natural breaks (jenks) were applied to the remaining 11 vegetative and anthropogenic grids. Jenks are designed to best group similar values and maximize differences between classes by placing breaks at relatively large jumps in data values. The 10 breaks corresponded to the relative number of grid cells, which were used as a proxy for habitat suitability. Higher numbers of grid cells received a higher score, which was considered equal to habitat with a higher species-specific preference or suitability and a lower cost of movement through it. A score of 1 corresponded to values that had the fewest number of grid cells compared with a score of 10 that had the highest number of grid cells. While relative scores (1–10) were kept consistent among species, the specific break values shifted on a species-by-species and grid-by-grid basis.

For each species, these 12 reclassified grids were combined into a weighted overlay for a final resistance or cost surface. The grids were weighted equally; therefore, each frequency grid (n = 11) was equal to 8% of the total and the single categorical land use grid was equal to 12% of the total. Each species was then analyzed independently for LCP and LCC; however, in each case the LCP was used to help “center’ the corridor on areas with minimal resistance but maximal connectivity between the protected areas in the N-C zones. Protected areas in each zone were set as source and destination sites in both analyses, which allowed the smaller protected areas south of Parque Provincial (PP) Urugua-í and north of Reserva de Biósfera Yabotí/PP Esmeralda to be used as “stepping stones” (Fig 1).

### Evaluation of cost analyses

While Rabinowitz et al. [20] used the lowest 0.10% of grid values (or cell values) in the LCC as the cutoff for area to be included in determining corridors in a wide-range model for jaguars, this level was determined to be insufficient at the small scale in this study because it prevented connectivity with the largest protected area in the central region, Reserva de Biósfera Yabotí/PP Esmeralda. For example, among the five species, the oncilla and bush dog achieved complete connectivity with this area when the cutoff was set to 0.3% and 0.4%, respectively. When the cutoff was expanded to include the lowest 0.15% and 0.20% of grid values, connectivity with this area was achieved; specifically, via a northern (0.15% and 0.20%) and northwestern (0.20%) connection. However, this also resulted in an expansion in the breadth of area included in the corridor; therefore, this study used the LCP and home range information to determine the optimal balance between corridor length and width [10,11,19]. We aimed to make the species-specific corridors wide enough to support sufficient territories for these wide-ranging species [11], while keeping the total area at a level that could be feasibly implemented. We allowed flexibility in the species-specific width of the corridor, however, aimed to maintain a minimum width of 14 km around the LCP, values that mimicked the maximum potential home range values were used to establish predictor value grids (Table 1).

With an overlay of the five species-specific LCP-LCC models, it was possible to narrow the overall width of the corridor and define two multispecies corridors that could be used to set conservation priorities: a primary (1°; 7 km) and a secondary (2°; 14 km). Together, both multispecies corridors range between 1–3× the minimal home range (50 km^2^) of a jaguar, puma, and bush dog, with some areas in the combined 1°- 2° corridor extending beyond 3× the minimal home range.

The quality of area in these multispecies corridors was evaluated by quantifying the overlap with the species-specific ENMs, PSR, and degree of habitat fragmentation/modification. In addition, we quantified the overlap of habitat type with PSR level. Areas that crossed through protected areas (i.e., currently managed) were eliminated, so final values reflect land needing priority management. Together these data identified areas within the 1° and 2° corridors that need protection of current habitat and those that need habitat restoration.

### Quantifying the effect of roads

We evaluated PSR and habitat quality relative to roads that cross the 1° and 2° corridors and identified areas needing additional attention to minimize road kills. All major national and provincial roads, dirt and paved (Fig 1), were included since many dirt roads are currently undergoing conversion to asphalt and others have had this change proposed. Any relationship between roads and PSR or habitat is not an artifact of these models because the ENMs did not include road frequency or distance. To quantify potential effect of roads, three buffers were applied to roads that fell into the 1° and 2° corridors: 200 m, 500 m and 1 km total width. We determined the proportion of the PSR levels and habitats by quantifying the amount that intersected each buffer relative to the total area of the buffer. For example, the number of cells with a PSR of five species in the 100 m road buffer of the 1° corridor was divided by the total number of cells in the 100 m buffer of the 1° corridor.

### Evaluation of multispecies corridor vs single-species corridor

The 1° and 2° multispecies corridors were evaluated against two sets of jaguar data allowing us to determine if these multispecies corridors captured the jaguar’s restricted ecological needs. First, the overlap of PSR and the jaguar’s ENM were quantified for both corridors. Second, we quantified the overlap between the multispecies corridors, PSR, and jaguar’s ENM with areas that Schiaffino et al. [59] defined as core areas and main corridors in the jaguar’s landscape. These core areas were subdivided by Schiaffino et al. [59] based on priority and protection: high priority requiring protection (JC1), intermediate priority requiring protection (JC2), intermediate priority functioning as buffers (JC3), and low priority (JC4). We quantified the overlap of these four subdivisions with the two multispecies corridors.

## Results

### Final ENM evaluation

**Fig 2:**
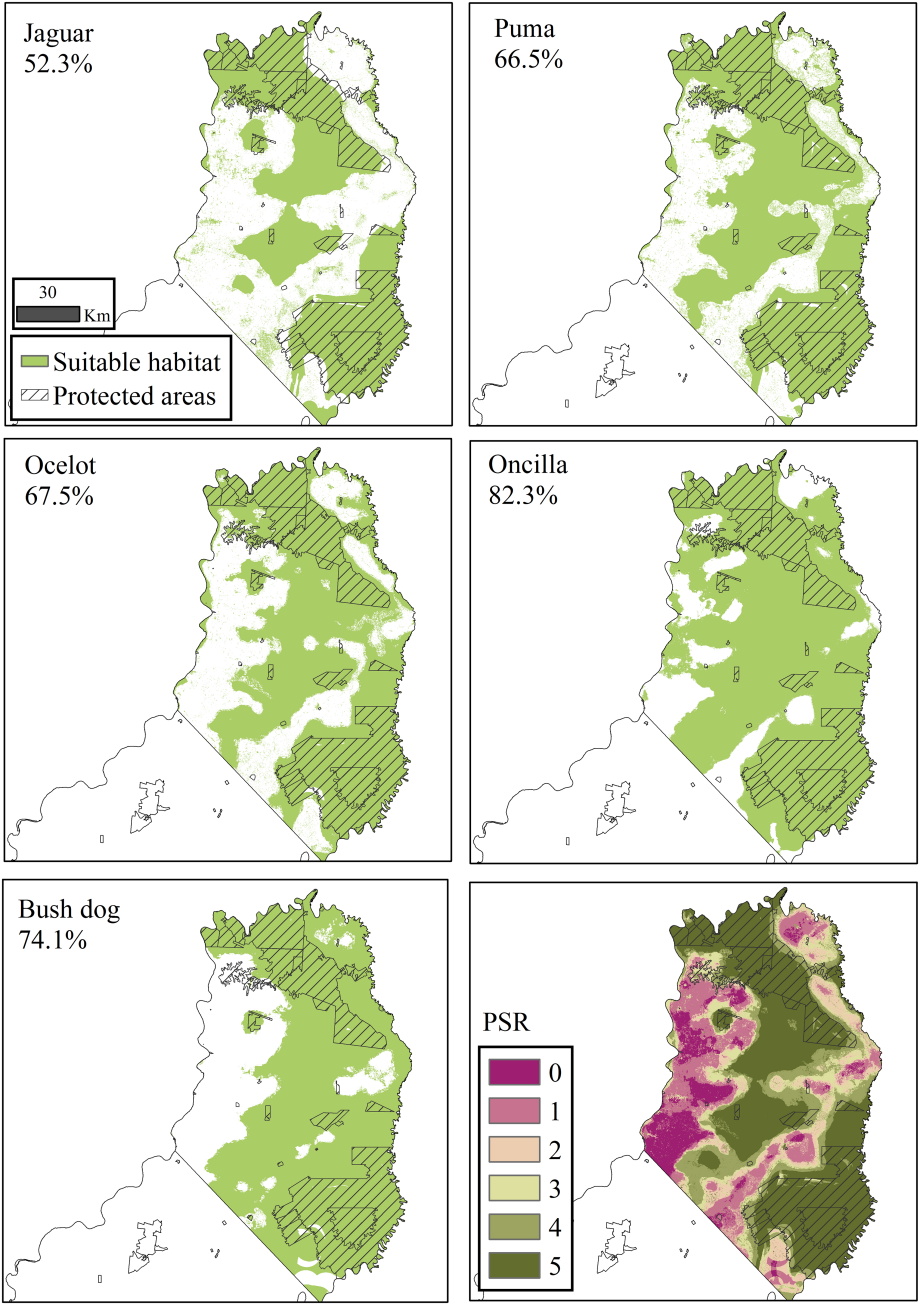
The five species-specific ecological niche models with % suitable habitat across the northern-central zones noted and the potential species richness (PSR) that quantifies the overlap (0–5 species) among those areas defined as suitable for the five carnivores.

The AUC values were ≥ 0.85 for test data (jaguar = 0.934±0.020 (SD), puma = 0.851±0.045, ocelot = 0.864±0.037, oncilla = 0.850±0.015, bush dog = 0.859±0.075) indicating a high accuracy in discriminating areas of species’ presence and absence [60]. When threshold values were assigned, the jaguar was identified as the most restricted with only 52.3% of the total area defined as suitable habitat, which contrasts with the other four carnivores (range = 62.1–82.3%; Fig 2 and Table 2). Despite this range in proportion of suitable habitat among the five carnivores (52.3%-82.3%), all had similar quantities of forest in their potential distributions (595,245.1–861,774.3 ha) with a <10% spread or difference; however, puma, oncilla, and bush dogs had slightly higher levels of modified habitats compared to jaguar and ocelot (Table 3).

**Table 2:** The amount and proportion of suitable habitat, as determined with species-specific ecological niche models, across the northern-central (N-C) zones in Misiones, Argentina, the 1° corridor, and the 2° corridor. The N-C zones ALL refers to the total amount (hectares) and proportion (%) of suitable habitat across the N-C zones of Misiones including overlap with existing protected areas. In contrast, the N-C Zones OUTSIDE PA excludes areas that overlap with existing protected areas. The 1° corridor and 2° corridor refers to the total amount and proportion of suitable habitat that overlaps with the corridor excluding areas that overlap with existing protected areas.

	N-C zones ALL		N-C zones OUTSIDE PA		1° corridor		2° corridor	
	Suitable area	% total	Suitable area	% total	Suitable area	% total	Suitable area	% total
**Jaguar**	788,000.5	52.3	383,504.3	48.7	137,329.6	61.5	209,386.1	53.4
**Puma**	1,003,103.6	66.5	566,505.1	56.5	197,854.9	88.6	316,793.2	80.8
**Ocelot**	1,017,353.6	67.5	577,981.2	56.8	200,710.6	89.9	317,962.7	81.1
**Oncilla**	1,240,363.7	82.3	797,860.3	64.3	216,149.6	96.8	366,392.6	93.4
**Bush dog**	1,116,487.4	74.1	683,852.0	61.3	204.794.1	91.7	339,848.0	86.7

**Table 3:** Proportion (%) of habitat relative to the total area defined as suitable in the species-specific ecological niche models.

	Jaguar	Puma	Ocelot	Oncilla	Bush dog
**Forest**	77.2	74.4	79.8	70.7	75.1
**Tree plantations**	9.9	9.6	8.7	10.8	7.3
**Agriculture**	5.6	8.5	4.2	8.3	7.7
**Mixed use**	2.5	3.2	3.5	5.5	6.1
**Urban**	2.1	1.6	1.3	1.9	<1.0
**Pasture**	<1.0	1.0	1.1	1.7	2.2

### Potential species richness

These similarities and differences among the five carnivores in the amount and type of habitat in their potential distributions (Table 3) help explain the PSR (Table 4). Together these five ENMs capture the overlap and unique characteristics of the five carnivores as indicated by the finding that the area occupied by the highest PSR levels (≥4 species; 59.4%) is like the mean amount of suitable habitat across the five ENMs both with (68.5%) and without (57.5%) protected areas included.

**Table 4:** Proportion (%) total area compared across the range of potential species richness (0–5 species) for the northern-central (N-C) zones in Misiones, the 1° and 2° corridors, and the mean for the three road buffers (200 m, 500 m, and 1 km) in both corridors.

				Road buffers	
	N-C zones	1° corridor	2° corridor	1° corridor	2° corridor
**0 species**	7.8	0.3	0.9	0.8	2.5
**1 species**	13.9	3.5	8.1	7.3	13.3
**2 species**	9.8	5.8	8.5	9.5	13.1
**3 species**	9.1	8.2	10.4	8.4	11.6
**4 species**	15.3	22.1	21.5	19.3	17.3
**5 species**	44.1	60.1	50.6	54.7	42.2

### Evaluation of cost analyses

**Fig 3:**
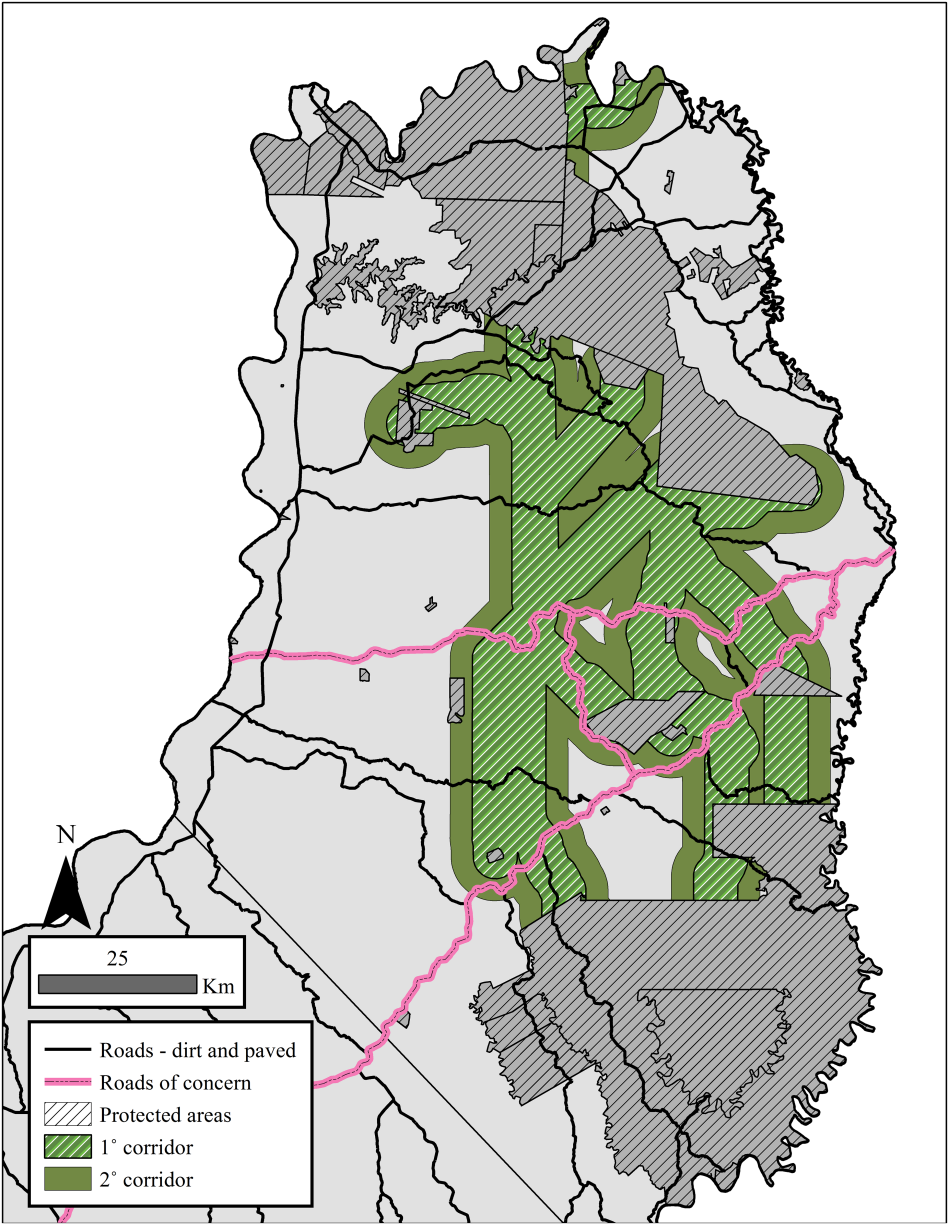
The defined multispecies corridor between protected areas in the northern-central zones of Misiones, Argentina. The corridor was narrowed and divided into two levels that could be used to set conservation priorities: a 1° (7 km width) and a 2° (14 km width) corridor.

The 1° and 2° corridors contain 223,236.4 ha and 392,104.1 ha, respectively. These areas represent regions lacking formal protection, since all area intersecting protected areas was excluded from the corridor analyses. This fact, combined with the overlap between the two corridors in eastern Misiones (Fig 3), explains why the 2° corridor does not have twice the total area as the 1° corridor.

While the 1° and 2° corridors hold similar proportions of suitable habitat for the five carnivores, the proportion is slightly higher in the 1° corridor (61.5–96.8%) compared to the 2° corridor (53.4–93.4%; Table 2). The corridors effectively maximize the capture of suitable habitat for all five carnivores as seen in the higher proportion in either corridor compared to that outside of protected areas in the N-C zones (48.7–64.3%; Table 2); however, the jaguar was again the most restricted as is reflected in the lowest proportion of suitable habitat in the 1° and 2° corridors compared to the other four carnivores (Table 2).

While PSR ranged from 0–5 species, 50–60% of area in the corridors contained the five carnivores and this area increased by ~20% when PSR was expanded to include ≥4 species (Table 4). As with the species-specific ENMs, the higher PSR in either corridor versus the values found across the N-C zones indicated that the corridors effectively maximized capture of high species overlap or areas suitable for multiple species (Table 4).

Native forest versus modified habitats occupied the majority (>60%) of area in both corridors (Table 5). The similarity in the proportion of native forest occupying both corridors and the mean amount of native forest across the five ENMs (75.4%) indicated that the corridors effectively captured the native forest located outside of protected areas (1° = 29.8% and 2° = 48.2%).

**Table 5:** Proportion (%) of native forest and modified environments for the northern-central (N-C) zones in Misiones, the 1° and 2° corridors, and the mean for the three road buffers (200 m, 500 m, and 1 km) in both corridors.

				Road buffers	
	N-C zones	1° corridor	2° corridor	1° corridor	2° corridor
**Forest**	61.9	67.3	61.9	42.2	35.5
**Tree plantations**	13.0	13.2	14.1	12.0	14.5
**Agriculture**	10.7	8.2	10.1	18.7	20.2
**Mixed use**	6.1	5.9	7.0	12.0	13.6
**Pasture**	3.0	1.7	2.4	5.1	6.0
**Bare ground**	1.9	1.7	2.3	5.2	5.0
**Water**	0.5	---	---	---	---
**Urban**	1.0	0.3	0.4	1.8	2.2
**Campos/grasslands**	---	---	---	---	---
**Unclassified**	1.9	1.7	1.8	3.0	3.0

When the quality of habitat (native versus modified) in both corridors was quantified across three PSR levels (≤3 species, 4 species, and 5 species), two patterns were found: 1) a positive relationship between PSR level and proportion of native forest and 2) a negative relationship between PSR level and proportion of modified habitat (Table 6).

**Table 6:** Proportion (%) of native forest and modified environments (with amount of unclassified area removed) in the 1° and 2° corridors at three levels of potential species richness.

	1° corridor			2° corridor		
	≤3 species	4 species	5 species	≤3 species	4 species	5 species
**Forest**	47.5	66.2	75.4	41.0	64.0	74.8
**Tree plantations**	8.9	11.4	15.6	14.8	11.1	15.5
**Agriculture**	20.6	10.0	4.1	20.6	11.5	4.1
**Mixed use**	14.2	8.5	2.6	13.8	8.5	2.8
**Pasture**	5.3	2.0	0.6	5.8	2.0	0.7
**Bare ground**	3.5	1.5	1.4	4.0	2.0	1.6
**Water**	---	---	---	---	---	---
**Urban**	---	0.4	0.3	---	0.9	0.5
**Campos/grasslands**	---	---	---	---	---	---

**Fig 4:**
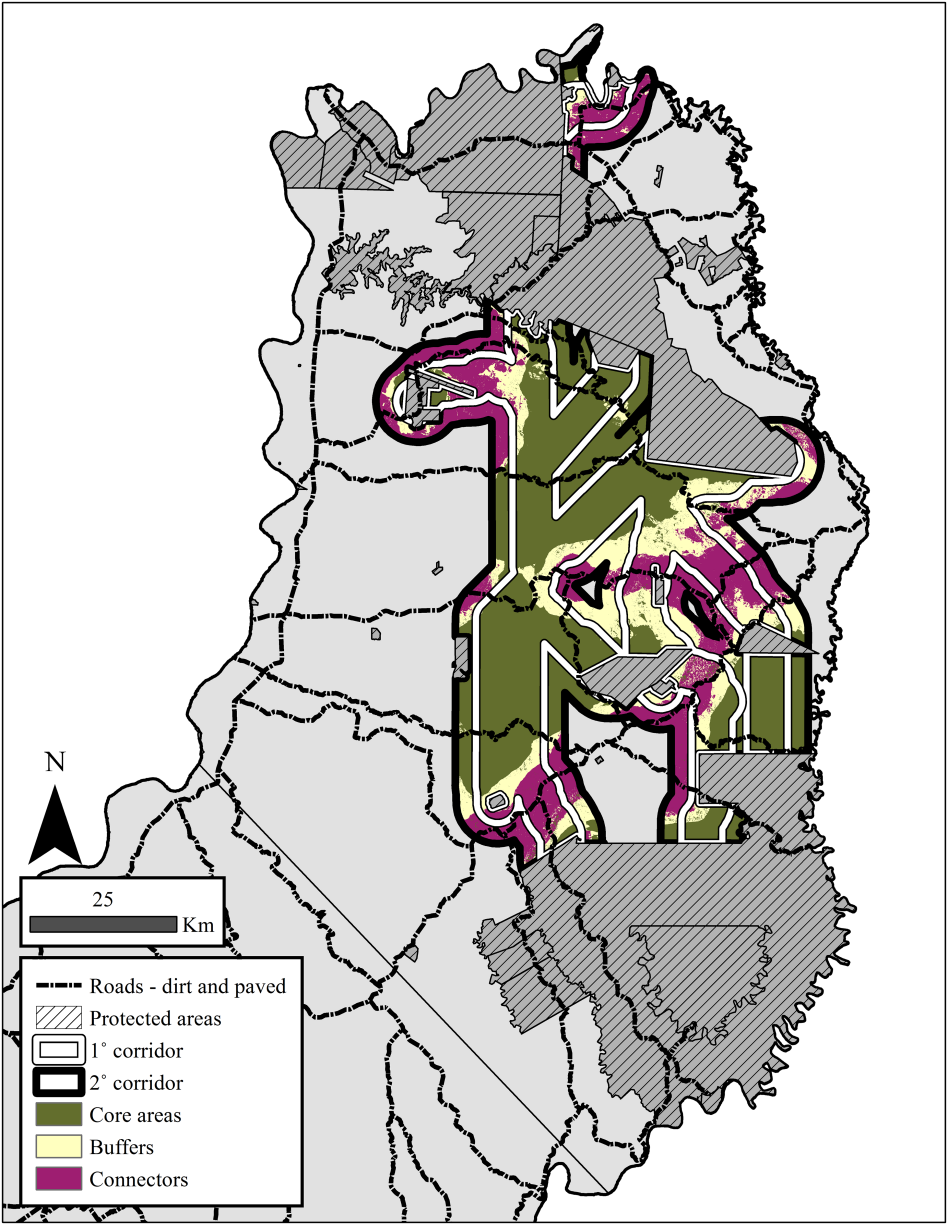
Map showing the three key areas (core, buffers, and connectors) in the 1°and 2° multispecies corridors that were identified as needing protection or varying degrees of habitat restoration. These classifications are based on the level of potential species richness (low, moderate, or high) combined with the current level of habitat integrity.

Combining these evaluations of cost analyses allowed us to divide the 1° and 2° multispecies corridors into three areas needing protection and varying degrees of habitat restoration: 1) core areas needing protection to maintain high PSR and high levels of native forest, 2) buffers located around these core areas needing protection to minimize habitat conversion so moderate PSR levels can be maintained and possibly increased in the future, and 3) connectors where low PSR levels can be potentially increased through protection that minimizes habitat conversion and habitat restoration that increase native forest (Fig 4). While both the core areas (~75%) and buffers (~65%) have high levels of native forest, the connectors contain <50% with the remainder split primarily among agriculture (~20%), monoculture tree plantations, mixed use areas and pastures. Since all areas in the corridors are outside of formal protected areas, the day-to-day monitoring will be the responsibility of the land owners with official actions against poaching or habitat destruction requiring cooperative agreements with provincial park guards and federal police. In both the 1° and 2° corridors, core areas occupied most of the area (134,192.4 ha and 198,469.0 ha) with buffers (49,234.32 ha and 84,270.8 ha) and connectors (39,809.6 ha and 109,364.3 ha) sharing almost equal extensions.

### Quantifying the effect of roads

The 2° corridor contained twice as many major roads (442.5 km) compared to the 1° corridor (216.1 km). Because a low variability was found across the three buffers at each PSR and habitat type in both the 1° and 2° corridors, results were simplified by calculating the mean at each value (Tables 4 and 5). Near roads there was a shift towards a higher proportion of low species richness and higher proportion of altered habitat compared with values across each corridor (Tables 4 and 5).

In the southwest-northeast region and west of Reserva Privada (RSP) Aguaraí-mi, this negative effect of roads on PSR and habitat type (Fig 1) is likely more dramatic than these calculations indicate because the lack of a strong negative effect in the northern, western, and southern portions likely mutes the total effect. This difference between the regions probably reflects the increasing network of roads, habitat conversion, and expanding rural populations in the former regions. There are three primary roads with areas of special concern in the southwest-northeast region: east-west provincial road (RP) 17, northeast-southwest national road (RN) 14, and north-south RP20 (Fig 1).

### Evaluation of multispecies corridor vs single-species corridor

Both corridors are effectively capturing the ENM of the highly restricted jaguar, with almost identical proportions found between the ENM of the jaguar and the highest PSR in the 1° and 2° corridors (Tables 2 and 4). The jaguar’s potential distribution in the 1° and 2° corridors has ≤5% overlap with areas that have ≤4 species (Table 4) reinforcing the fact it is the most restricted species since its distribution is almost exclusively limited to areas where the other four species occur.

The jaguar’s ENM and PSR in the 2 multispecies corridors were overlaid on the core areas in the jaguar’s landscape defined by Schiaffino et al. [59]. The jaguar’s core areas had a ~50% overlap with the 1° and 2° corridors and much of this overlapping area had a high PSR of either four (15.7% and 17.1%) or five (79.1% and 75.1%) species. These core areas also had a strong alignment (~70% overlap) with the jaguar’s ENM.

In the 1° and 2° corridors, almost 100% of the jaguar’s main corridors [59] were contained in the areas with ≥4 species. As was found across the five carnivores, these main corridors are primarily composed of native forest (~75%) with plantations as the second largest proportion (~12%).

When the four subdivisions of the jaguar’s core areas [59] were overlaid on the 1° and 2° corridors, JC1 (>60%) and JC2 (>30%) had the highest degree of overlap. When combined, these two subdivisions composed almost 100% of the overlap (>97%). In the areas of overlap in both the 1° and 2° corridor, much of the four subdivisions had a high PSR (4–5 species).

## Discussion

The multispecies approach used in this study emphasized the potential importance of expanding beyond a single umbrella or focal species when developing biological corridors that aim to capture the varied ecological requirements of coexisting species and ecological processes across the landscape. Our multifaceted cost analysis developed corridors that maximized the connectivity between protected areas in the N-C zones of Misiones, Argentina, while ensuring these corridors were centered on areas with the lowest ecological cost for the five carnivores. Despite variation in body size, the jaguar, puma, ocelot, oncilla, and bush dog overlap in their ecological requirements; however, this is not without variation in the degree of habitat flexibility [8,29,61,62]. Incorporating variation in anthropogenic measures across the landscape in species-specific ENMs (Table 1), allowed us to differentiate the combination of factors that constrain the suitable habitat for each species [8,28,29] (Table 2). Puma, oncilla, and bush dog have comparatively higher levels of modified habitats in their potential distributions compared to the jaguar and ocelot. This variation is mirrored in the range of suitable habitat among the five carnivores (52.3%-82.3%) and the proportion of area that contains the highest PSR (≥4 species; 59.4%). Using these species-specific preferences, we assigned costs across the heterogeneous landscape and used the overlap of species-specific cost analyses to model the multispecies corridor. The application of these techniques is not unique to this region for many reasons, including the fact that ongoing habitat fragmentation is affecting the movement and habitat connectivity in ecosystems worldwide plus the distribution of these five carnivores expands beyond the boundaries of Misiones, Argentina.

In addition to balancing the needs of the five carnivores, the final model balanced the overall dimensions of the corridors, since protecting over 500,000 ha between the two zones is not realistic or feasible. The use of LCPs allowed for central routes between the N-C zones to be identified. Despite the restrictions that required the width and length of the corridor to be balanced, we could reach the minimal width recommended by previous studies [11] with a final corridor that was a 1–3× the minimum home range of the carnivores, with some areas in the corridor having a width that extended beyond this. The evaluation of the cost analyses demonstrated that the multispecies corridors effectively capture and maximize the amount of suitable habitat for all five carnivores, especially when compared to spread and distribution of potential values across the N-C zones. Based on Gilbert-Norton [63] the investment in conserving this area would be worthwhile for the carnivores since the corridor connects existing suitable habitat in a heterogeneous landscape versus using man-made structures as connectors.

Our secondary analysis that focused on the unique requirements of the highly restricted jaguar ensured that we effectively captured the needs of this endangered species. First, the multispecies corridors were compared to corridors designed specifically for the jaguar [59]. An overlap with primarily high species richness and strong overlap with the jaguar’s ENM from this study indicated the jaguar’s core areas defined by Schiaffino et al. [59] were effectively captured. Similarly, the almost 100% overlap of the main corridors defined by these authors, which were composed primarily of native forest, indicated they were effectively captured in the multispecies corridors. Second, the multispecies corridors were compared with the jaguar’s ENM from the current study. The jaguar’s >95% overlap with the other four carnivores demonstrated that, while it had a very restricted potential distribution and habitat requirements, both were completely captured in the broader distributions and habitat requirements of the other four carnivores. This finding illustrates the benefit of using multiple species versus a single species to develop corridors, because using only the highly restricted jaguar to develop the corridor would mean that the potential distributions of the other four carnivores would be restricted and decreased by ~20–30%. Therefore, while the jaguar-specific model effectively captured the needs of that single species, it failed to completely capture the varied ecological requirements of coexisting species and ecological processes across the landscape; however, the multispecies model balances the needs of the five carnivores [10,11]. So, it appears that at least in Misiones province, the jaguar should not be modeled as an umbrella species because the results fail to capture the varied requirements of coexisting species across the breadth of potential habitats. Caution should be applied in similar ecosystems where ongoing habitat fragmentation may result in extreme movement restrictions for species like jaguar, which have been shifted to limited areas due to their preference for specific habitat types.

In the 1° and 2° corridors, we could differentiate areas that need varying degrees of protection or habitat restoration: core areas, buffers, and connectors (Fig 4). While core areas and buffers contain the highest levels of species richness and suitable habitat, the connectors cannot be ignored. Even though these connectors only compose ~15–25% of the total area in the corridor, they are essential to maintain complete connectivity between the N-C zones. The three core areas of high concern are regions where buffers and connectors intersect in the corridors: 1) the northeastern portion between PP Urugua-í and PP Piñalito, 2) the central portion between PP Cruce Caballero/Valle del Arroyo Alegría and Reserva de Biósfera Yabotí /PP Esmeralda, and 3) the central portion between RSP Yaguarundí and Reserva de Biósfera Yabotí /PP Esmeralda. While core areas, buffers, and connectors need to have minimal habitat conversion, expansion of roads, and new human settlements, the connectors also require efforts towards habitat restoration so that the amount of suitable habitat and species richness can be increased in these areas. These three core areas of high concern correspond to areas where roads have a negative effect on habitat quality and species richness, with areas of special concern associated with RP17, RN14, and RP20 in the southwest-northeast region. More detailed studies using sign of animal activity (e.g., road kills, sightings, tracks, scat) along roads would help determine optimal locations for wildlife crossings under/over roadways [64,65,66].

Misiones is not like some areas where feasibility can be directly linked to the economics underlying the ability to acquire or purchase the land in the corridor [67], as the land is almost exclusively in private hands. Therefore, when prioritizing parcels in the corridor to target, it is important to acknowledge that intersecting the corridor does not mean that the private land is completely contained in either corridor; instead, only a portion of the parcel may overlap. While this provides the option to target only portions of a property for conservation, it also provides other options that can benefit efforts to maximize coverage within and across the corridors. Specifically, expansion of a parcel beyond the “boundaries” providing a natural connection between core areas, buffers, and connectors, a natural expansion from the 1° to the 2° corridor, or a way to maximize the width and extend beyond the 2° corridor. This type of expansion may be especially important in situations where it is not possible to develop a 1°and 2° corridor that share such similar composition and dimensions as we had. In these cases, the ability to expand beyond boundaries in selected locations would allow capture of suitable habitat to maximize corridor width. Once the general area of the corridor is identified, specific properties can be narrowed based on size and adjacency of parcels, owners with multiple properties contained in the corridor, current activities on the property, and expressed landowner interest. These factors allow the number of parcels targeted to be minimized while maximizing the coverage across the corridors.

The approach in making a corridor a reality is multipronged and involves a strong investment from the local community especially when developing corridors that use existing protected areas as “stepping stones”, as private land will inevitably be involved to varying degrees in and around the corridor. One important conservation action should involve developing an effective way to communicate findings and future efforts with the public, including printed materials and online information. This will allow one to gain direct feedback, questions, and concerns from the people living in and around the corridor. Another conservation action should involve local training programs with a goal to establish a network of professionals able to assume all aspects related to the long-term monitoring and management of the established corridor. We know that LCP/LCC approach used in this study can provide a framework that can integrate new field data [20], allowing additional work to optimize management strategies, direct protection efforts, and maximize long-term of local biodiversity. In addition to the long-term conservation impacts within Misiones, this comprehensive approach can serve as a model to other regions that show habitat loss and lack of data. First, the techniques used in this study allowed multiple species to be surveyed across varied habitat types independent of the degree of legal protection. The ability to collect this data efficiently and rapidly, and then use GIS technology to model much needed conservation strategies, means there is a way to implement long-term changes in other regions of the world. Specifically, the multifaceted cost analyses developed here balanced the trade-offs of single and multispecies corridors while maximizing connectivity among existing protected areas. Second, by making training a network of local conservationists a basic component in establishing a corridor, one can help to ensure the corridor’s long-term success. Third, since the species in this study have ranges across the Neotropics the results from this study can be expanded to understand connectivity between countries, with analyses at the species or community level. This expands into examining the overlap among the predators and their prey, the effect of poaching, and areas of concern for long-term survival of either taxon.

## Supporting information

S1 Appendix Details of the 761 scat swabs with confirmed species identity. For the 727 felid samples and 34 bush dog samples, the location and zone (North-Central) are summed by species. For protected areas, the total area is reported in parentheses.
(PDF)
