# Supplementary material for: Using niche-modelling and species-specific cost analyses to determine a multispecies corridor in a fragmented landscape
Source: PLoS One. 2017 Aug 25;12(8):e0183648. doi: 10.1371/journal.pone.0183648 (PMC5571917; doi:10.1371/journal.pone.0183648)
Supplement: S1 Appendix — For the 727 felid samples and 34 bush dog samples, the location and zone (North-Central) are summed by species. For protected areas, the total area is reported in parentheses. (PDF) [file pone.0183648.s001.pdf]

S1 Appendix. Details of the 761 scat swabs with confirmed species identity. For the 727 felid samples and 34 bush dog samples, the location and zone (North-Central) are summed by species. For protected areas, the total area is reported in parentheses.

**Location**	**Zone**	**Jaguar**	**Puma**	**Ocelot**	**Oncilla**	**Bush dog**
Ejército Argentino (6,951 ha)	N	2	2	---	6	2
Parque Nacional Iguazú (54,380 ha)	N	24	13	27	---	---
Parque Provincial (P.P.) Guardaparque H. Foerster (4,309 ha)	N	---	---	6	6	1
P.P. Puerto Península (6,900 ha)	N	9	4	3	52	5
P.P. Urugua-í (84,000 ha)	N	13	9	17	37	---
Refugio Privado Aguaraí-mi (3,050 ha)	N	---	---	---	7	---
Reserva Nacional Iguazú (12,620 ha)	N	---	---	---	3	---
Reserva Natural Privada Yate-í (15 ha)	N	---	---	---	7	---
Reserva Privada Karadya (90 ha)	N	---	---	---	4	---
Reserva San Jorge (21,163 ha)	N	5	3	5	22	1
Reserva Yacutinga (539 ha)	N	---	---	11	---	---
P.P. Araucaria (92 ha)	C	---	---	---	1	---
P.P. Cruce Caballero (522 ha)	C	---	2	1	2	---

Valle del Arroyo Alegría (8,000 ha)	C	---	1	---	39	1
P.P. Cruce Caballero (522 ha) & Valle del Arroyo Alegría (8,000 ha)	C	---	---	---	2	---
P.P. Esmeralda (31,569 ha) & Reserva de Biósfera Yabotí (236,313 ha)	C	2	5	16	43	3
P.P. Piñalito (3,796 ha)	C	---	---	---	9	3
Reserva Privada Yaguaroundí (400 ha)	C	---	---	---	4	---
outside protected areas	N	8	9	22	106	17
outside protected areas	C	---	11	3	144	1
		**63**	**59**	**111**	**494**	**34**
